# Pb‐Pb Dating of Terrestrial and Extraterrestrial Samples Using Resonance Ionization Mass Spectrometry

**DOI:** 10.1029/2020EA001177

**Published:** 2020-10-23

**Authors:** F. Scott Anderson, Carolyn Crow, Jonathan Levine, Tom J. Whitaker

**Affiliations:** ^1^ Southwest Research Institute Boulder CO USA; ^2^ Department of Geological Sciences University of Colorado Boulder Boulder CO USA; ^3^ Department of Physics and Astronomy Colgate University Hamilton NY USA

**Keywords:** chronology, Pb‐Pb, Moon, Mars

## Abstract

We are developing an in situ, rock‐dating spectrometer for spaceflight called the Chemistry, Organics, and Dating EXperiment (CODEX). CODEX will measure Rb‐Sr compositions and determine ages of samples on the Moon or Mars and can be augmented to obtain Pb‐Pb ages. Coupling Rb‐Sr and Pb‐Pb measurements broadens the suite of samples that can be dated and could provide tests of concordance. Here we assess whether geochronologically meaningful Pb‐Pb data could be measured in situ by tuning the prototype CODEX to acquire Pb‐Pb data from a suite of well‐characterized specimens from the Earth, Moon, and Mars. For Keuhl Lake Zircon 91500 our ^207^Pb/^206^Pb age of 1,090 ± 40 Ma is indistinguishable from the accepted age. In each of the Martian meteorites we studied, we could not resolve more than a single component of Pb and could not uniquely determine ages; nevertheless, our isotopic measurements were consistent with most previous analyses. On the other hand, we uniquely determined ages for three lunar meteorites. Our age for MIL 05035 is 3,550 ± 170 Ma, within 2σ of published ages for this specimen, in spite of it having <1 ppm Pb. LAP 02205 was contaminated by terrestrial Pb, but by filtering our data to exclude the most contaminated spots, we obtained an age of 3,010 ± 70 Ma, coincident with published values. Finally, our age for NWA 032 is nearly 1,000 Ma older than its age determined from other isotopic systems and is supported by additional Pb measurements made after chemical leaching.

## Key Points

- We have measured Pb in meteorites from the Moon and Mars, and a terrestrial zircon, using a prototype dating instrument for spaceflight
- Our instrument reproduces dates for the zircon and two lunar samples, and our Mars results are consistent with previous Pb studies
- Our NWA 032 result is the first Pb‐Pb isochron age for this sample but is unexpected (3.76 Ga), and discordant with other isotopic system

## 1 Outstanding Problems in Solar System Chronology

Five decades after the Apollo astronauts returned to Earth with the first lunar samples for detailed study, the absolute chronology of the Moon—and, by extension, of the inner solar system—continues to be rife with uncertainties on multiple timescales. Though many lunar samples have been radioisotopically dated, only a few of their dates are securely associated with specific geologic settings on the Moon. The few contextualized and dated samples are the tie points for chronologies based on crater counting, which are used to estimate the ages of terranes on the Moon and other planetary bodies. The crater‐density chronology is unconstrained during the interval between 3,000 and 1,000 Ma (Hartmann, 1966, 1970; Hartmann et al., 2000; Neukum, 1977; Neukum et al., 1975; Stöffler & Ryder, 2001) because none of the samples of known provenance collected on the Moon have been dated to this period. Crater counts derived from Lunar Reconnaissance Orbiter images suggest that Apollo‐era chronologies might be miscalibrated by up to 1,100 Myr during this interval (Robbins, 2014). Dating a new suite of lunar samples from localities with the appropriate crater density can resolve this discrepancy, and dating precision of hundreds of millions of years is sufficient to do so.

A second critical issue in planetary science is the timing and duration of the giant basin‐forming impacts on the Moon. Tera et al. (1973, 1974), and Turner et al. (1973) were the earliest to note the great preponderance of 4,000–3,800 Ma ages among the Apollo samples and argued for a “cataclysm” of giant impacts over that relatively brief interval. There are at least 11 basins that are stratigraphically assigned to the Nectarian Period (Fassett et al., 2012), and yet Maurer et al. (1978) found that the first and last of these occurred only about 100 Myr apart (~3,980–3,880 Ma), based on ^39^Ar‐^40^Ar dating of Apollo 16 samples. However, some authors argued against the cataclysm hypothesis from the outset (e.g., Hartmann, 1975), and a number of recent studies (e.g., Boehnke & Harrison, 2016; Conrad et al., 2018; Michael et al., 2018; Zellner, 2017) have also disputed the evidence for it. Much of the debate is focused on the extent to which the ages of lunar samples in our collection, including both Apollo samples (e.g., Zhang et al., 2019) and meteorites (e.g., Gnos et al., 2004), should be associated with the Imbrium impact (Fernandes et al., 2013; Schaeffer & Schaeffer, 1977). This critical uncertainty regarding the existence of a lunar cataclysm will need to be addressed by a new mission specifically to collect samples undisturbed by Imbrium. Nectaris is a particularly worthy goal, because crater density and stratigraphy imply that it is particularly old; therefore, comparing its age with that of Imbrium will determine the full duration of the Nectarian Period, encompassing all the basin‐forming impacts stratigraphically assigned to it by Fassett et al. (2012). If Nectaris were created by an impact as recently as 3,900 Ma (James, 1982), then the 11 basins of the Nectarian Period must have formed quickly, in less than about 200 Ma. On the other hand, if Nectaris is as old as 4,250 Ma (Schaeffer & Husain, 1974), then those giant impacts occurred less than half as often, and the Nectarian impact rate was only half as “cataclysmic.” Moreover, a young Nectaris implies a relatively quiet pre‐Nectarian period from 4,400–4,000 Ma and demands that a new population of impactors must have been delivered to the inner solar system in a late heavy bombardment (e.g., Ryder, 2002); an old Nectaris favors models in which giant basin formation is consistent with a steady decline in impact rate at the end of planetary accretion. Meaningfully distinguishing between the endmember possibilities will require 1σ dating precision of ~75 Ma, so that an “old” age of 4,250 Ma, a “young” age of 3,900 Ma, and an intermediate age can be resolved with 95% confidence. Collection of additional samples to determine other basin ages would permit us to move beyond testing endmember hypotheses and to trace the giant basin formation rate through time. Even finer dating precision will enable more detailed studies of important geologic processes on the Moon and other planetary bodies. These might include identifying the sequential lava flows that form maria on the Moon, or the ages of lunar irregular mare patches, or timing the progressive desiccation of a formerly more habitable Martian valley. Any of these experiments would demand a mobile, in situ dating instrument, which could acquire samples from multiple localities in a field site, and analyze them swiftly enough to help drive subsequent sample collection.

## 2 In Situ Geochronology

In addition to being able to provide scientific results during a mission, an in situ dating instrument has great advantages of cost and risk over a sample return mission. It is unrealistic to imagine the resources being allocated for the fleet of sample‐return missions are sufficient to address the wealth of science questions that would merit them. In situ dating experiments on the surfaces of solar system bodies therefore will be essential for establishing the absolute timing of geologic processes throughout the history of the solar system (National Research Council, 2011).

In fact, though it has long been assumed that obtaining meaningful ages for specimens from the Moon or Mars would require sample‐return missions, the era of in situ dating has already begun. Farley et al. (2014) and Martin et al. (2017) used three different instruments aboard the Curiosity rover on Mars to measure the concentrations of elemental K and Ar isotopes in samples encountered in situ. On the assumption that all the ^40^Ar they observed was radiogenic, they calculated plausible K‐Ar ages for two mudstones, with ~300 Ma statistical precision. Vasconcelos et al. (2016) described some of the difficulties of obtaining reliable and self‐consistent rock ages using the Curiosity instruments, which were not designed for geochronology.

By contrast, several independent efforts have worked to ready purpose‐built in situ dating instruments for future spaceflight missions (e.g., Anderson et al., 2012, 2013; Cattani et al., 2019; Cohen et al., 2014; Riedo et al., 2013). Such instruments boast considerably better statistical precision (e.g., Anderson et al., 2020; Cho & Cohen, 2018) than the age measurements made so far on Mars. More importantly, their ability to analyze concentrations of both radioisotopic parents and radiogenic daughters in multiple spots on a sample allows for the calculation of isochron ages. That is, direct observation of a correlation between the concentrations of parent and daughter isotopes intrinsically tests whether the specimen is consistent with a single age at all, or whether the bulk concentrations have been affected by the vagaries of geologic processes operating at multiple episodes in the long history of the solar system. In this way, isochron dating techniques remove a potentially large systematic error in the interpretation of geochronological data.

## 3 Chemistry, Organics, and Dating EXperiment

We have developed and built a prototype in situ instrument for isochron dating, called the Chemistry, Organics, and Dating EXperiment (CODEX), a laser‐ablation resonance ionization mass spectrometer. The instrument consists of an ablation laser that vaporizes ~100 μm spots from a specimen's surface, lasers tuned to resonantly excite and ionize atoms of selected elements, and a time‐of‐flight mass spectrometer that extracts, separates by mass, and detects the photoions. These components can be combined in different ways to enable a wide range of analyses. Each pulse of intense light from the ablation laser creates a short‐lived vapor plume consisting of neutral atoms, ions, molecules, and some polyatomic clusters. Admitting the ions into the time‐of‐flight analyzer allows us to collect laser‐ablation mass spectra, which reflect the chemical composition of a specimen. In other modes of operation, we electrostatically repel those ions away from the mass spectrometer to interrogate other constituents of the plume. Our dating work focuses on the neutral atoms of elements in a radioactive parent‐daughter relationship; the resonance lasers irradiate the plume with light whose wavelengths match the energy gaps between each element's atomic ground state and an excited state, and between that excited state and a higher one. Having excited the elements of interest to high‐lying energy states, we further irradiate the plume with an infrared laser that ionizes only atoms close to their ionization thresholds. The photoions created in this way are then analyzed by the mass spectrometer. We can also use our infrared lasers without their wavelength‐tuned counterparts to obtain two‐step laser mass spectra of organic molecules, which we demonstrated on a sample of the Murchison meteorite (Anderson et al., 2014).

Resonance ionization is a versatile technique that can be applied to most elements in the periodic table. Our previous dating efforts with CODEX have employed the ^87^Rb‐^87^Sr decay system, which we selected in part because Rb‐Sr is more robust to alteration than other dating systems. Specifically, a survey (Anderson et al., 2015a) of the Mars Meteorite Compendium (Meyer, 2011) shows that ^87^Rb‐^87^Sr ages are supported by agreement with ages from other isotopic systems more often than K‐Ar, the other dating system currently under development for portable dating. Using CODEX, we measured Rb‐Sr ages for the Martian meteorite Zagami (Anderson et al., 2015a) and the Duluth Gabbro (Anderson et al., 2015b), both with precision better than a 200 Ma spaceflight goal (National Research Council, 2015). More recently, we have achieved 20 Ma precision on Zagami (Anderson et al., 2020). Though still finer precision can be achieved by laboratory‐based instruments, 20 Ma uncertainty is sufficient to settle the billion‐year question of lunar cratering history, and even to resolve the hundreds‐of‐millions‐of‐year endmember uncertainties in the timing and duration of giant basin formation.

Laboratory development of CODEX began in 2008. The data presented in this paper were acquired using our benchtop instrument, described in detail in Anderson et al. (2015a, 2015b) Currently, the benchtop instrument has seven lasers, sufficient to measure Rb‐Sr or other isotope pairs like Pb‐Pb, but cannot measure Rb‐Sr and Pb‐Pb, for example, at the same time. Work began on the spaceflight model of CODEX in 2014, based on hardware that has already been developed for flight (e.g., our mass spectrometer), or actually flown (like the sample handling system), with current efforts focusing on space hardening its laser systems. The spaceflight laser systems we are presently developing for ablation and Rb‐Sr resonance ionization are master oscillator fiber amplifiers, which can produce 100–200 μJ (266, 776, 780, 461, and 497 nm) to 1 mJ (1,064 nm) of laser light in nanosecond pulses at a repetition rate of 10 kHz. The power supplies and timing electronics in our instrument are already small enough to be portable. We have a flight design for a much smaller mass spectrometer (Scherer et al., 2006; Wurz, Abplanalp, Tulej, Iakovleva, et al., 2012; Wurz, Abplanalp, Tulej, & Lammer, 2012). The flight instrument will be roughly 50 cm on each side and have a mass of 50 kg, including all the lasers, similar to the size and 40 kg mass of the Surface Analysis at Mars (SAM) instrument on the Mars Science Laboratory rover (Mahaffy et al., 2012). However, unlike SAM, CODEX has no moving parts and no consumables other than power. Miniaturized fiber lasers for resonance ionization of Sr and Rb are nearing completion, though one is being redesigned to improve thermal stability and power efficiency. These lasers have a repetition rate of 10 kHz and so will allow us to acquire data 500 times faster than we do at present.

## 4 An Additional Chronometer for CODEX

The geochronological interpretation of isotopic data becomes much stronger when multiple isotopic systems yield concordant ages, so we focus in the present paper on adding the analytical capability of an additional isotopic system to CODEX. An obvious candidate would be the ^40^K‐^40^Ar geochronometer, which is also under development for spaceflight (e.g., Cattani et al., 2019; Cho & Cohen, 2018; Cho et al., 2016; Cohen et al., 2014; Devismes et al., 2016; Solé, 2014). This dating system, embracing both K‐Ar and ^39^Ar‐^40^Ar techniques, is among the most powerful tools for dating terrestrial, lunar, Martian, and meteorite samples (Jourdan et al., 2014; McDougall & Harrison, 1999). From the perspective of CODEX, however, Ar is impossible to analyze selectively by resonance ionization mass spectrometry, because the photons required to promote electrons from the ground state to even the lowest‐lying excited state are so energetic that they could nonresonantly ionize other species as well. Therefore, introducing a K‐Ar capability would require the incorporation of entirely new hardware. Furthermore, the relative mobility of Ar presents dating challenges in certain cases; for example, trapped argon can make a sample appear erroneously old (e.g., Bogard & Garrison, 1999; Bogard & Park, 2008), and episodes of open‐system behavior to noble gases can make a sample look erroneously young (e.g., Boehnke & Harrison, 2016; Fleck et al., 1977).

Alternatively, the U‐Pb geochronometer is another powerful tool widely used to date terrestrial rocks, the Earth, and extraterrestrial samples including meteorites (Allegre et al., 1995; Amelin, 2006; Amelin et al., 2002, 2005; Borg et al., 2011, 2015; Hopkins & Mojzsis, 2015; Murthy & Patterson, 1962; Nier et al., 1941; Norman & Nemchin, 2014; Patterson, 1953, 1955, 1956; Patterson et al., 1955; Snape et al., 2018). This geochronometer incorporates two decay systems, ^238^U‐^206^Pb and ^235^U‐^207^Pb. The existence of the two decay systems together allows an age to be determined from isotopes of Pb alone (for theoretical details of Pb‐Pb dating, see Connelly et al., 2017), which is advantageous for CODEX because (1) interelement fractionation is absent and (2) only a single set of resonance lasers tuned to electronic transitions in Pb would be required, rather than needing a set of lasers for U as well.

Pb‐Pb dating has been used extensively in planetary science. Patterson (1956) and Patterson et al. (1955) were the first to propose a 4.5 billion age for the solar system when they observed that terrestrial and meteorite samples comprised a single Pb‐Pb isochron. More recently, Pb‐Pb dating has become the technique of choice for obtaining precise absolute ages of calcium‐aluminum‐rich inclusions (e.g., Bouvier & Wadhwa, 2010), chondrules (e.g., Amelin & Krot, 2007), and ancient meteorites (e.g., Connelly et al., 2008). Pb isotopes have been used to date samples from both the Moon (e.g., Crow et al., 2017; Hopkins & Mojzsis, 2015; Liu et al., 2012; Nunes et al., 1973, 1974; Premo et al., 1999; Tatsumoto, 1970; Tatsumoto et al., 1977; Tatsumoto & Rosholt, 1970; Tera & Wasserburg, 1972a, 1972b, 1972c) and Mars (e.g., Borg et al., 2005; Bouvier et al., 2005, 2008, 2009; Chen & Wasserburg, 1986) and also to constrain the geochemical evolution of the samples' parent materials (e.g., Bellucci et al., 2018; Borg et al., 1999; Snape et al., 2016). Notwithstanding the widespread applicability of Pb‐Pb dating for extraterrestrial samples, the trend in terrestrial geochronology has been away from dating using Pb measurements alone. Advances in U‐Pb dating of zircons (Mattinson, 2005), whose U/Pb ratios are so high that the challenge of instrumental fractionation of elements is outweighed by the precision achievable on large U signals, has meant that Pb‐Pb dating is less commonly applied to terrestrial rocks where sample quantities are abundant enough to find zircons.

Two pitfalls of the Pb‐Pb technique bear mentioning. The first is that Pb isotope ratios can lie along linear arrays that result in spurious isochron “ages” if a sample behaved as an open system during any of its history. While closed‐system behavior is a pervasive assumption in radioisotope dating, Pb‐Pb isochrons are unlike isochrons from other systems in that this assumption can be violated without necessarily causing Pb isotope ratios to deviate from a linear array (Amelin, 2006). The second is the issue of sample contamination by external sources of Pb. This has been a particular problem for Martian meteorites and, to a lesser degree, the lunar meteorites. Unlike the Moon, Mars has a low U/Pb ratio (e.g., Borg et al., 2005), so the small quantities of radiogenic Pb in Martian samples can easily be overwhelmed by the addition of anthropogenic Pb (e.g., Patterson, 1965), or even by secondary Martian Pb (Bellucci et al., 2016). Contamination with terrestrial Pb poses the reverse problem for lunar samples: The U/Pb ratio of the bulk Moon is so high (e.g., Premo et al., 1999) that the Pb in contaminated samples can carry too little inherited lunar Pb to constrain the isotopic composition of this component.

Though terrestrial Pb contamination will have affected the measurements we report here on meteorites, clean preparation of the spaceflight instrument can ensure that it does not affect measurements CODEX would make in situ on Mars or the Moon. Introducing Pb from a meteorite's environment, whether on Earth or Mars, can result in a ternary mixture of radiogenic, inherited, and environmental Pb that makes it difficult to constrain the isotopic composition of the radiogenic endmember (e.g., Premo & Tatsumoto, 1992). In the case of in situ analyses, the issue of terrestrial common Pb will be removed, which would in theory result in better isochrons. However, even in the event of ternary mixing with some previously unidentified Pb reservoir, it may be possible to use ages derived from other isotopic systems such as Rb‐Sr, coupled with Pb isotopic observations to constrain important geochemical signatures such as the isotopic evolution of mantle and crustal reservoirs (e.g., Bellucci et al., 2018). Thus, endowing CODEX with both Rb‐Sr and Pb‐Pb capability could yield scientific benefits in geochemistry as well as geochronology.

In summary, the Pb‐Pb system is an excellent candidate for precise in situ dating on another planetary body, and the science return could be especially high if it were used in tandem with Rb‐Sr. Though in this study we had only enough lasers to resonantly excite isotopes of Pb, we stress that if lasers tuned to Rb and Sr were also at hand, we could obtain isotopic data for all three elements simultaneously.

## 5 Samples

Here we report a first CODEX test of Pb‐Pb dating on a suite of samples from the Earth, Moon, and Mars. All of these samples have been environmentally exposed to anthropogenic Pb. A future experiment will endeavor to validate CODEX by analyzing Apollo samples from the Moon that have been carefully curated to avoid terrestrial Pb contamination.

In this study, we analyzed a Zircon 91500 from Keuhl Lake, Ontario (Wiedenbeck et al., 1995); the Martian meteorites Zagami (Treiman & Sutton, 1992) and Northwest Africa 7034 (Agee et al., 2013); and the lunar meteorites Miller Range 05035 (Joy et al., 2008), LaPaz Icefield 02205 (Anand et al., 2006), and Northwest Africa 032 (Fagan et al., 2002). Each sample was cut with a Buehler ISOMET 5000 precision saw to ~8 mm × 8 mm × 5 mm, so that it would occupy approximately half of our sample holder, and it was attached with vacuum epoxy to a similarly sized cut slab of a standard. The stub was hand‐polished to create a flat surface for analysis, with sample and standard faces coplanar, and then sonicated in methanol to remove contaminant and cross‐contaminant dust from the surface. Different standards were mounted alongside each sample, as noted below.

### 5.1 Kuehl Lake Zircon 91500

We were provided with a slice of 91500 zircon provided to us by D. Trail of the University of Rochester. This widely used reference is derived from a large syenite pegmatite crystal from near Kuehl Lake, Ontario, with a ^207^Pb/^206^Pb thermal ionization mass spectrometry (TIMS) age of 1,065.4 ± 0.63 Ma (1σ, MSWD = 1.3), Pb abundance of ~15 ppm, and U abundance of ~81 ppm (Ibanez‐Mejia et al., 2015; Schoene et al., 2006; Wiedenbeck et al., 1995). Our sample was ~25 mm^2^ in area. The zircon measurements were calibrated against NIST standard SRM‐612, using the preferred values from the GEOREM database (Jochum et al., 2005) for known isotope ratios.

### 5.2 Martian Meteorites: Zagami and NWA 7034

In order to assess our ability to date Martian meteorites, we purchased a 0.313 g cut slab of the fine‐grained lithology of Zagami, measuring ~3 mm × 7 mm × 8 mm, from E. Twelker at the Meteorite Market, and were loaned ~2 cm^2^ sample of NWA 7034 by C. Agee of the University of New Mexico.

The basaltic shergottite Zagami has been dated using TIMS of leachates and mineral separates (Borg et al., 2005; Bouvier et al., 2005; Chen & Wasserburg, 1986). Rb‐Sr and Sm‐Nd ages have been determined by Shih et al. (1982), Nyquist et al. (1995), and Borg et al. (2005), and all these studies report ages between 160 and 180 Ma. This young age has been explained variously as the time of crystallization (Borg et al., 2005), impact ejection of the meteorite from Mars (Nyquist et al., 1995), or the last contact of the sample with acidic fluids in the Martian subsurface (Bouvier et al., 2005).

The Pb‐Pb isotope systematics in Zagami have been the subject of much debate (e.g., Bellucci et al., 2016; Borg et al., 2005; Bouvier et al., 2005). Borg et al. (2005) studied U and Pb in Zagami and argued for severe disturbance of the U‐Pb system in the meteorite. Though they noted a linear trend among Pb isotopes, which, taken at face value, would have implied a ~4,000 Ma age, their preferred explanation for the linear array of data was as a mixing line between the initial Pb in Zagami and contaminating terrestrial Pb. On the other hand, Bouvier et al. (2005) argued that the lack of terrestrial Pb contamination in nakhlites is inconsistent with a mixing explanation for the Zagami data and instead favors the interpretation of a true 4,048 Ma isochron age. Bellucci et al. (2016) concurred with Borg et al. (2005) in the interpretation of mixing but proposed that the contaminant is derived from a globally distributed Pb reservoir on Mars.

Very radiogenic Pb in Zagami has been detected by Zhou et al. (2013), who analyzed baddeleyite grains by secondary ion mass spectrometry and showed that the radiogenic Pb was consistent with the age deduced from the Rb‐Sr and Sm‐Nd systems. Whereas the more abundant minerals in Zagami, and other Martian meteorites, develop very little radiogenic Pb because of the generally low U/Pb ratios of the Martian mantle, baddeleyites concentrate U when they crystallize and therefore are especially valuable for detecting radiogenic Pb in Martian meteorites (Moser et al., 2013). Baddeleyite is also a decomposition product of zircon in high shock environments and therefore may potential be useful for dating impact events if the U‐Pb systems are reset during the transformation (El Goresy, 1965; Wittmann et al., 2006).

The spot size of our ablation laser is ~80 μm, considerably larger than the typical baddeleyite grains studied by Zhou et al. (2013) in Zagami (~20 μm). Therefore, our measurements cannot probe the radiogenic Pb carried by the baddeleyites without also sampling the terrestrial and inherited Pb carried by other phases. For the present experiment, we sought to learn whether we could notice any of our spot analyses carrying the signature of the radiogenic Pb preserved in the baddeleyites, even when mixed with surrounding minerals. We used the same piece of Zagami that we have analyzed in our Rb‐Sr dating experiments, most recently obtaining an age of 150 ± 20 Ma (Anderson et al., 2020). Our Zagami measurements were calibrated with USGS standard GSD‐1G, using the preferred values from the GEOREM database (Jochum et al., 2005) for known isotope ratios.

NWA 7034 is a porphyritic basaltic breccia with feldspar and pyroxene phenocrysts (Agee et al., 2013) and is thought to be paired with NWA 7533 (Humayun et al., 2013). We included this specimen in our study, in spite of the fact that it consists of clasts potentially of diverse origins, in order to assess the suitability of CODEX to analyze the many brecciated rocks likely to be found in situ on Mars or the Moon. While breccias are certainly not ideal for multiphase isochron dating, even on the millimeter scale, it may not be possible for an in situ dating mission to garner a more ideal sample or to isolate zircons for single‐crystal analyses. Zircons from NWA 7533 have been dated to 4,428 ± 25 Ma (Humayun et al., 2013), with evidence of a later disturbance at 1712 ± 85 Ma (1σ; MSWD = 2.4). The bulk rock Pb abundance is ~6 ppm, and the U abundance is ~0.4 ppm (Humayun et al., 2013). Bellucci et al. (2016) argue that NWA 7533 represents a previously unrecognized reservoir of Martian Pb and that this reservoir may have mixed with Pb signatures from other SNC meteorites with young Rb‐Sr and Sm‐Nd ages, resulting in unexpectedly older Pb‐Pb dates (Bellucci et al., 2016). Our NWA7034 measurements were calibrated with NIST standard SRM‐612, using the preferred values from the GEOREM database (Jochum et al., 2005) for known isotope ratios.

### 5.3 Lunar Meteorites: LAP 02205, MIL 05035, and NWA 032

In order to assess our ability to date lunar meteorites, we obtained an ~2 cm^2^ sample of NWA 032 from R.A. Langheinrich Meteorites and were loaned ~50 mm^2^ samples of LAP 02205 and MIL 05035 through the Johnson Space Center Curation and Analysis Planning Team for Extraterrestrial Materials (CAPTEM) program. The meteorites LAP 02205, MIL 05035, and NWA 032 are potentially paired (Liu et al., 2009) and thought to be associated with REE‐rich western nearside lunar terranes (Anand et al., 2006).

MIL 05035 is a coarsely crystalline (~5 mm grain size), pyroxene‐ and plagioclase‐rich, low‐Ti mare basalt meteorite found in Antarctica and likely derived from a KREEP‐rich terrane. SIMS U‐Pb measurements of U‐rich zirconolites indicate an age of 3,851 ± 8 Ma (2σ; MSWD = 1.4) (Zhang et al., 2010), consistent with Sm‐Nd, Rb‐Sr, and Ar‐Ar measurements (Fernandes et al., 2009; Nyquist et al., 2007). The bulk‐rock abundances in MIL 05035 of Pb (0.39–0.42 ppm) (Liu et al., 2009) and U (0.07 ppm) (Joy et al., 2008) are low, so to our knowledge, no one has attempted whole‐rock Pb‐Pb dating before. Our MIL 05035 measurements were calibrated against USGS standard BHVO‐2, using the preferred values from the GEOREM database (Jochum et al., 2005) for known isotope ratios.

LAP 02205 is a crystalline low‐Ti lunar basaltic meteorite dominated by pyroxenes and plagioclase. LAP 02205 was found in Antarctica and has a similar major element composition to Apollo 12 and 15 mare basalts, as well as to NWA 032 and MIL 05035 (Borg et al., 2009). SHRIMP U‐Pb dating of phosphate minerals in LAP 02205 indicates an age of 2,929 ± 150 Ma (2σ; MSWD = 1.04) (Anand et al., 2006). The degree of shock‐induced maskelynitization of plagioclase is lower than in other mare basalts, suggesting the date is relatively undisturbed. Nyquist et al. (2005) measured an Rb‐Sr age of 3,020 ± 30 Ma, and an Ar‐Ar age of 2,950 ± 20 Ma. Likewise, Rankenburg et al. (2007) reported a Rb‐Sr age of 2,991 ± 14 Ma and a Sm‐Nd age of 2,992 ± 85 Ma. The abundance of Pb is ~1 ppm, and that of U is ~0.55 ppm (Anand et al., 2006). Our LAP 02205 measurements were calibrated with USGS reference glass GSD1‐G, using the preferred values from the GEOREM database (Jochum et al., 2005) for known isotope ratios.

NWA 032 is an unbrecciated low‐Ti mare basalt with a groundmass of pyroxene and plagioclase containing phenocrysts of olivine, pyroxene, and chromite intermixed with terrestrial weathering products (Borg et al., 2009; Fagan et al., 2002; Zeigler et al., 2005). U and Pb abundances are ~0.45 and ~1 ppm, respectively (Barrat et al., 2005). Neither Pb‐Pb nor U‐Pb measurements of NWA 032 have been previously reported; however, TIMS, combined with progressive leaching, reveal concordant Rb‐Sr and Sm‐Nd ages of 2,947 ± 16 Ma and 2,931 ± 92 Ma (Borg et al., 2009). Fernandes et al. (2003) reported a younger Ar‐Ar age of 2,779 ± 14. We analyzed NWA 032 in two separate runs, once comounted with NIST SRM‐612 and once with BHOV‐2, in both cases using the preferred values from the GEOREM database (Jochum et al., 2005) for known isotope ratios of the standards.

## 6 Laser Ablation Resonance Ionization of Pb

**Figure 1:**
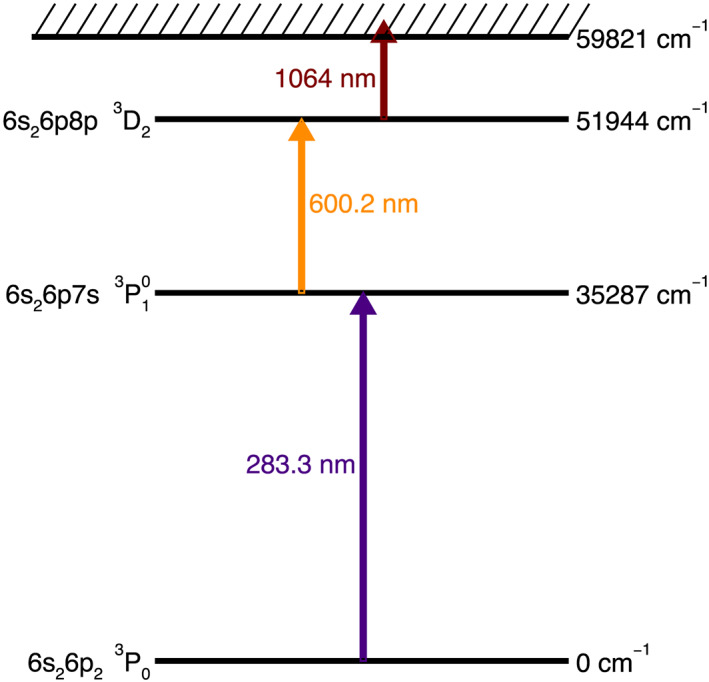
Resonance excitations of Pb used in this paper.

The CODEX instrumental approach has been previously described in Anderson et al. (2015a, 2015b) and Anderson et al. (2020) and is only summarized here, though with additional discussion of the modifications and improvements used for Pb‐Pb. CODEX uses laser ablation (~5 ns pulses of 213 nm radiation into ~80 μm spots, ~0.6 GW/cm^2^) to vaporize a small bit of the target rock, generating ions, neutral atoms, and a few polyatomic clusters. The ions are electrostatically rejected from entering our time‐of‐flight mass spectrometer. About 1 μs after ablation, lasers tuned to 283.3 and 600.2 nm are used to resonantly excite electrons of neutral Pb atoms in the vaporized plume, and the atoms in the resulting excited states are photoionized with a 1,064 nm laser (Figure 1). The photoions travel back and forth through the multibounce mass spectrometer before being detected by an SGE ETP hybrid discrete dynode detector. We acquire data from thousands of ablation pulses to analyze each of hundreds of spots on a sample, allowing us to determine a Pb‐Pb isochron age using isotopic data from different minerals, in a manner analogous to the 3 to 20 analyses normally made in a TIMS experiment.

We analyzed our samples in much the same way as we previously analyzed the Duluth Gabbro and Zagami (Anderson et al., 2015a, 2015b). We began each new spot analysis by conditioning the sample surface with 3,000 ablation pulses in order to expose fresh sample material by removing the layer that would have experienced the most chemical exchange with the environment. Next, we collected 4,000 shots of data, alternating between groups of 100 pulses with the resonance lasers on and 100 pulses with the resonance lasers off, producing a pit <100 μm deep. Data with resonance lasers off correspond to background from ions produced nonselectively by the ablation process. Both for resonance pulses and background pulses, we removed shots that were too large for the digitizer to capture, and we checked for and corrected detector saturation and baseline offsets. Next, we calculated peak areas, standard errors, and covariances for ^204^Pb, ^206^Pb, ^207^Pb, and ^208^Pb. We retained in subsequent analysis all spots in which the three abundant Pb isotopes were detected at >2σ level above the background, but ^204^Pb signals were often too small to enforce a similar condition. We analyzed the comounted standard before and after every set of four sample spot analyses, and we used the standard data to calculate instrumental fractionation factors. To interpret isochron diagrams of the ^207^Pb/^206^Pb ratio versus the ^204^Pb/^206^Pb ratio, we assumed values for the ^238^U/^235^U ratio of 137.786 ± 0.011 (Connelly et al., 2012, 2017), and the activity constants *λ*_238_ = 1.55125 × 10^−4^ Myr^−1^ and *λ*_235_ = 9.8485 × 10^−4^ Myr^−1^ (Villa et al., 2016a, 2016b).

There are two noteworthy improvements in this experiment relative to our previous work. First, we had noticed that ablation laser pointing varied by tens of microns over the duration of a spot analysis. Because our ablation spot size is about 80 microns, these translations were sufficient to effectively sample portions of the specimen that had not undergone the surface conditioning performed at the beginning of each measurement. To address this issue, we added a Thorlabs beam profiler to monitor the pointing of a partial reflection of the ablation beam, and a computer‐driven, three‐axis piezo‐adjustable mirror to compensate for drifts in beam pointing. The resonance beams do not suffer from the same issue because their beam diameters are large (~5 mm) relative to the beam‐pointing changes.

Second, we improved the synchrony of the resonance lasers. Though we had already controlled absolute timing of the Q switches to within 400 ps using Highland Technologies T560 digital delay generators, we had nevertheless observed drifts of several nanoseconds among the arrival times of the resonance laser pulses. Delays of this magnitude are comparable with the lifetimes of the electronic states we seek to excite, so having lasers this far out of synchrony leads to reduced efficiency for the resonance ionization process and can introduce isotopic fractionation. To ensure that the pulses arrived within 1–2 ns of one another, we implemented a set of high‐speed photodiodes, which monitor the arrival times, and used their outputs to adjust the timings of the laser Q switches.

## 7 Results

The data we report in this paper were all acquired during a campaign between March and June 2016 (Anderson et al., 2020). Our interpretation of the data has been aided by more recent advances in our understanding of Pb isotopic abundances in planetary materials (e.g., Bellucci et al., 2016, 2018; Connelly et al., 2017; Snape et al., 2016).

### 7.1 Sensitivity, Selectivity, and Odd‐Even Fractionation

We performed several experiments to characterize our implementation of resonance ionization for Pb. By observing the Pb signal size as we systematically varied the intensity of our resonance lasers, we found that we saturated the 283.3 nm transition at about 88 kW/cm^2^ (~50 μJ/pulse) and the 600.2 nm transition at about 530 kW/cm^2^ (~300 μJ/pulse). Saturation is important to mitigate any isotopic fractionation that could occur because of pulse‐to‐pulse fluctuations in laser intensity and wavelength. We acquired all our data thereafter with at least this much energy per pulse in each respective laser.

We verified that the Pb we were observing came from the sample rather than from the instrument itself by backing the specimen away from the focal point of the ablation laser and observing that the signal disappeared. Using NIST standard SRM‐612, which contains 0.55 ppm ^204^Pb, we observed a 3σ detection of ^204^Pb; this implies a detection limit for a given isotope of about 0.2 ppm. Additionally, we demonstrated the selectivity of our resonance ionization scheme by analyzing a standard with 14.9 ppm of ^208^Pb and 7.4 ppm of the potentially interfering isobar ^176^Hf^16^O_2_. The hafnium oxide does not have the same combination of electronic resonances characteristic of Pb, but one might imagine that the photon energy from either of our lasers could ionize it nonresonantly. However, we observed that switching off the 600.2 nm laser caused the signal at all Pb masses to disappear, even though the more energetic 283.3 nm photons were still present. This shows that essentially all of our signal comes from Pb atoms, which were excited by the double optical resonance, rather than from isobars.

We routinely observed fractionated ^207^Pb/^206^Pb ratios in standards, due to a well‐known odd‐even isotope effect in resonance ionization (e.g., Fairbank et al., 1989; Sankari, 2011). The odd‐even isotope effect is known to be very sensitive to laser wavelength. We found only weak dependence on the precise wavelength of the 283.3 nm laser, but significant sensitivity to the precise wavelength of the 600.2 nm laser. We monitored the wavelengths of both lasers and ensured that neither of these drifted by more than ~0.06 nm over the course of any run. We use the observed isotope abundances in standards to measure the instrumental fractionation as a function of time, and we correct our sample observations for this effect.

Finally, we measured Pb isotopes for the Keuhl Lake 91500 zircon, Zagami, NWA 7034, MIL 05035, LAP 02205, and NWA 032 (Table 1). One important source of uncertainty in all our analyses arose from the observation of somewhat noisy baselines in all our mass spectra. Though we have since learned to quiet the baseline by more effectively excluding ablation plasma from the mass spectrometer (Anderson et al., 2020), all our data reported here include a substantial baseline uncertainty. Propagation of this uncertainty is responsible for lower‐than‐expected mean square weighted deviances of our data from straight‐line fits.

**Table 1:** Samples

Parent body	Sample	Published ages (Ma)		This work			
		Other systems	Pb‐Pb	Matches published Pb‐Pb	Sufficient to determine age	Age (Ma)	Notes
Earth	Keuhl Lake Zircon 91500	1,062.4 ± 0.4 (U‐Pb)^a^	1,065.4 ± 0.3^a^	Yes	Yes	1,090 ± 40	Agreement at <1σ
Mars	Zagami	180 ± 4 (Rb‐Sr)^b^	4,048^e^	Yes	No	N/A	Observed mixture of inherited and terrestrial Pb. Radiogenic Pb component of Zhou et al. (2013) not observed.
		186 ± 5 (Rb‐Sr)^c^					
		183 ± 6 (Rb‐Sr)^c^					
		166 ± 5 (Rb‐Sr)^d^					
		180 ± 37 (Sm‐Nd)^c^					
		166 ± 12 (Sm‐Nd)^d^					
		155 ± 9 (Sm‐Nd)^e^					
		156 ± 6 (U‐Pb)^d^					
		230 ± 5 (U‐Pb)^f^					
		229 ± 8 (Th‐Pb)^f^					
		185 ± 36 (Lu‐Hf)^e^					
	Northwest Africa 7,034	2089 ± 42 (Rb‐Sr)^g^	4,114 ± 30^i^	Yes	No	N/A	Observed mostly inherited Pb, rather than ≥2 component mixing.
		2,190 ± 700 (Sm‐Nd)^g^					
		4,420 ± 70 (Sm‐Nd)^h^					
		4,426 ± 23 (U–Pb)^i^					
		4,367 ± 33 (U–Pb)^j^					
		1,574 ± 19 (U–Pb)^j^					
		4,411 ± 12 (U–Pb)^j^					
		4,311 ± 26 (U–Pb)^j^					
		1,357 ± 42 (U–Pb)^k^					
		4,439 ± 9 (U‐Pb)^l^					
Moon	Miller Range 05035	3,910 ± 6 (Ar‐Ar)^m^	3,851 ± 4^o^	Yes	Yes	3,500 ± 200	Agreement at <2σ
		3,845 ± 7 (Ar‐Ar)^m^					
		3,800 ± 50 (Sm‐Nd)^n^					
		3,900 ± 40 (Rb‐Sr)^n^					
	LaPaz Icefield 02205	2,985 ± 8 (Ar‐Ar)^m^	N/A	N/A	Yes	3,010 ± 70	Ternary mixing of inherited, terrestrial, and radiogenic Pb. Isochron age in <1σ agreement with other systems deduced after removing least radiogenic spots.
		2,889 ± 22 (Ar‐Ar)^m^					
		2,874 ± 28 (Ar‐Ar)^m^					
		3,020 ± 30 (Rb‐Sr)^p^					
		2,950 ± 20 (Ar‐Ar)^p^					
		2,929 ± 75 (U–Pb)^q^					
		2,991 ± 7 (Rb‐Sr)^r^					
	Northwest Africa 032	2,779 ± 14 (Ar‐Ar)^s^	N/A	N/A	Yes	3,760 ± 30	Pb‐Pb isochron age is much older than age from other systems.
		2,947 ± 16 (Rb‐Sr)^t^					
		2,931 ± 92 (Sm‐Nd)^t^					

^a^ Wiedenbeck et al. (1995).

^b^ Borg et al. (2005).

^c^ Shih et al. (1982).

^d^ Nyquist et al. (1995).

^e^ Chen and Wasserburg (1986).

^f^ Bouvier et al. (2005).

^g^ Agee et al. (2013).

^h^ Nyquist et al. (2016).

^i^ Bellucci et al. (2015) on paired meteorite NWA 7533.

^j^ Tartèse et al. (2014).

^k^ Humayun et al. (2013), on paired meteorite NWA 7533.

^l^ Yin et al. (2014).

^m^ Fernandes et al. (2009).

^n^ Nyquist et al. (2007).

^o^ Zhang et al. (2010).

^p^ Nyquist et al. (2005).

^q^ Rankenburg et al. (2007).

^r^ Anand et al. (2006).

^s^ Fernandes et al. (2003).

^t^ Borg et al. (2009).

### 7.2 Kuehl Lake 91500

We acquired 17 spot analyses on the Keuhl Lake 91500 zircon in just under 2 hr, before the ablation laser suffered a fault. Nevertheless, these analyses sufficed to constrain the age within 40 Ma.

**Figure 2:**
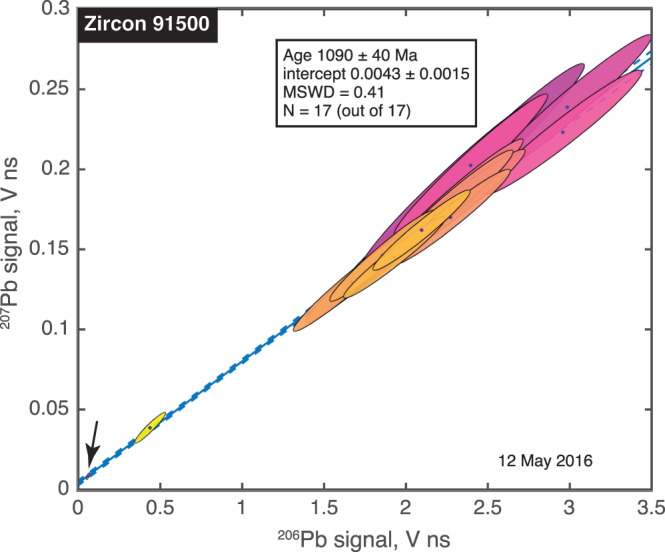
Pb‐Pb isochron for the Keuhl Lake 91500 zircon. Note well‐constrained ellipses near the origin, one of which is so small that we highlight it with an arrow. The accepted age is 1,065.4 ± 0.63 Ma (1σ, MSWD = 1.3) (Wiedenbeck et al., 1995) in agreement with our result of 1,090 ± 40 Ma. The uncertainties in absolute signal for ^207^Pb and ^206^Pb, which are large because of shot‐to‐shot fluctuations in ablation yield, are highly correlated, so that their ratio is much better constrained. In this and subsequent figures, 1σ uncertainty ellipses are shaded according to the precision of each measurement, from pink (greatest uncertainty) to yellow (greatest precision).

Because zircons so effectively exclude Pb when they form, the Pb found in zircons is almost purely radiogenic. Indeed, in 14 of 17 spots, the abundance of ^204^Pb, the only Pb isotope that is not radiogenic, was below our detection limit. Analyses of a single, isotopically homogeneous, radiogenic Pb component would yield ^207^Pb and ^206^Pb signals in perfect proportion and would plot on a straight line (i.e., an isochron) through the origin in Figure 2. We do observe a linear data array, whose slope of 0.0757 ± 0.0014 implies an age of 1,090 ± 40 Ma. This is consistent at the 1σ level with the published age of this sample (Wiedenbeck et al., 1995). Figure 2 shows that the absolute signal size for each isotope is usually quite uncertain but that the uncertainties are highly correlated, so the ^207^Pb/^206^Pb ratio is therefore precisely known. The leading source of uncertainty is shot‐to‐shot fluctuations in the ablation yield, which result in a variable number of Pb atoms of all isotopes in the ablation plume, but does not strongly fractionate isotopes one from another.

Interestingly, the best fit line through our data does not quite pass through the origin, suggesting that we detect a small admixture of nonradiogenic Pb together with the radiogenic Pb. This suggestion is supported by our detection of ^204^Pb in three of the spot analyses. The nonradiogenic Pb could either have been inherited by the zircon when it formed, acquired from the terrestrial environment, or a contaminant from sample handling in our lab. A strength of isochron dating is that correlating analyses from many spots on the sample allowed us to identify the presence of nonradiogenic Pb; without doing so, one might have estimated the age from the weighted mean of the measured ^207^Pb/^206^Pb ratios and, thus, obtained a value that would have been 80 Ma too old.

### 7.3 Analyses of Martian Meteorites

#### 7.3.1 Zagami

**Figure 3:**
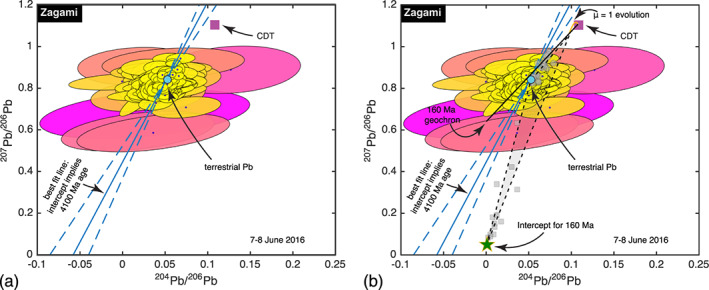
a. Pb‐Pb isochron diagram for Zagami, illustrating 190 spot analyses by their 1σ uncertainty ellipses. The data cluster near the terrestrial composition (Sutherland et al., 2003) but are slightly extended along a direction that suggests a ~4,100 Ma age. Also shown for reference is the Pb isotopic composition of Canyon Diablo troilite (CDT) (Tatsumoto et al., 1973), taken to represent the composition of primordial solar system Pb. Color code is described in Figure 2, b. As in Figure 3a but overlaid with published Pb isotopic analyses. Gray hexagons represent data from Chen and Wasserburg (1986), Borg et al., (2005), and Bouvier et al. (2005), along with phosphate analyses from Zhou et al. (2013). Baddeleyite analyses from Zhou et al. (2013) are plotted as gray squares. The shaded region bounded by dashed lines represents the ternary mixing field among Pb components near terrestrial composition (whether a contaminant from Earth or Mars), pure radiogenic composition with ~160 Ma age, and the composition expected for a reservoir with μ = ^238^U/^204^Pb nearly 1 having evolved from CDT until 160 Ma. Within the respective uncertainties, all of our data and all of the data from Zhou et al. (2013) are consistent with this field. Color code is described in Figure 2.

We analyzed 200 spots on Zagami over a 20‐hr period and detected ^206^Pb, ^207^Pb, and ^208^Pb above the 2σ confidence level in 190 of them. All 190 of these analyses are shown in Figure 3a. In 119 of the spots, even the low‐abundance isotope ^204^Pb was detected with greater than 95% confidence. Nevertheless, the largest uncertainty in most analyses stems from the uncertainty in the ^204^Pb abundance.

The ensemble of Zagami analyses clusters around the Pb isotopic composition of anthropogenic atmospheric Pb (Sutherland et al., 2003). Since we performed no leaching to try to remove terrestrial contamination, a simple interpretation of our data is that most of the Pb we observed in Zagami was incorporated since it fell to Earth in 1962 (McSween, 1985), either from the terrestrial environment or from the handling and preparing of the sample for analysis.

The cluster of analyses is somewhat extended along a line whose intercept would suggest a 4,100 ± 300 Ma age for the meteorite. In this regard, our data are quite similar to observations of Zagami by Chen and Wasserburg (1986), Borg et al. (2005), and Bouvier et al. (2005), all of whom applied chemical pretreatments to remove terrestrial Pb contamination (see Figure 3b). Chen and Wasserburg (1986) derived ^207^Pb/^206^Pb ages in excess of 4,000 Ma for Zagami leachates and residues, and Bouvier et al. (2005) interpreted their data to yield a 4,048 Ma isochron indistinguishable from the best fit line through our data. An alternative possibility was offered by Bellucci et al. (2016), who found Pb in several Martian meteorites beyond what could have been derived from in situ radioactive decay of U and Th. They proposed that this additional Pb was a contaminant from the Martian surface, which became embedded in the meteorites during ejection from Mars. According to Bellucci et al. (2016), the ~4,000 Ma “isochron” line is actually a mixing line between the contaminant Pb and Pb inherited when Zagami crystallized. The isotopic composition of the inherited Pb lies near the intersection of the ~160 Ma geochron and the evolution curve from primordial solar system Pb (Tatsumoto et al., 1973) for materials with relatively low μ = ^238^U/^204^Pb ratios (around 1). Our data permit explanation either as an isochron or as a mixing line.

Figure 3b shows that, like most other workers, we detected little or none of the radiogenic Pb component identified in baddeleyite grains by Zhou et al. (2013). One of the baddeleyite analyses overlaps with three of our spot analyses at the 1σ level, but our three analyses are also consistent with the best fit straight line through the entire ensemble of data. The baddeleyite analyses have fractional uncertainties up to 50%, so all of them are consistent with ternary mixing of contaminant, inherited, and radiogenic Pb. If we were to follow Borg et al. (2005) and use our Rb‐Sr analyses to *assume* an ~160 Ma age for Zagami, then we could use our data likewise to argue for consistency with mixtures of the same three components. But our direct detection of radiogenic Pb will require either smaller spot sizes, to sample small baddeleyites without the surrounding grains, or a larger set of spot analyses to capture one or more of these rare crystals. Neither of these pose difficult technical challenges; the ablation beam diameter could be narrowed with a pair of mirrors, and additional spot analyses simply take time.

#### 7.3.2 NWA 7034

**Figure 4:**
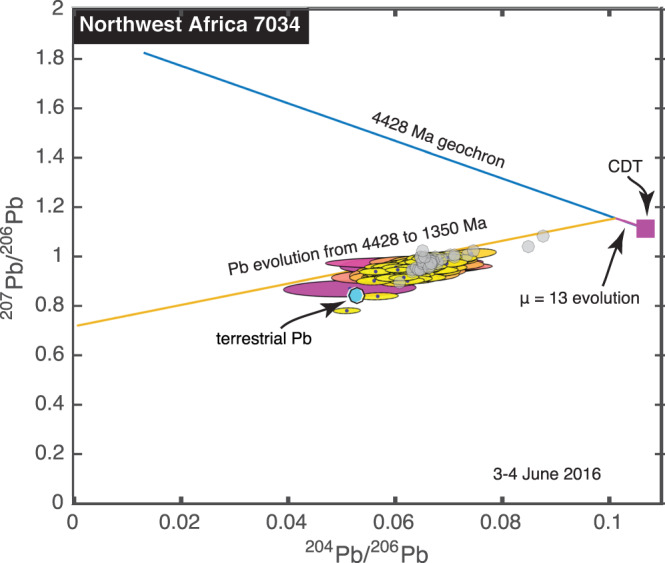
Pb‐Pb isochron diagram for NWA 7034. Our 200 measurements, represented by their 1σ uncertainty ellipses, form a single cluster whose center is decided offset from the terrestrial composition. Gray hexagons represent data from Bellucci et al. (2015) on Clasts 1, 4, 5, and 6 in the paired meteorite NWA 7533. Those workers proposed a model in which a Pb reservoir with μ = ^238^U/^204^Pb = 13 evolved from the primordial solar system composition (CDT) until 4,428 Ma and then gradually froze Pb compositions of phases, which were mixed together at 1350 Ma. Our data are consistent with that model but do not require it. Color code is described in Figure 2.

We spent 26 hr analyzing 200 spots on NWA 7034, and we detected the abundant isotopes ^206^Pb, ^207^Pb, and ^208^Pb in all of them. We also detected the low‐abundance isotope ^204^Pb at the >2σ level at 196 of the spots. All 200 analyses are shown in Figure 4.

The challenge of interpreting our data from this meteorite is that the 200 analyses form a single cluster, rather than being more spread out along a linear array. This was unexpected; since NWA 7034 is a breccia, a more likely prediction would have been for the Pb isotopic abundances to be scattered in a multidimensional field on an isochron diagram. Unlike the data from Zagami, this cluster of data from this meteorite is decidedly offset from the terrestrial Pb composition, though a small number of spot analyses could be consistent with anthropogenic Pb. Fitting a line through our data yields an age of 2,500 Ma, but the large 900 Ma uncertainty is a consequence of the low spread of the data points along any isochron line.

Bellucci et al. (2015) performed secondary ion mass spectrometry on Northwest Africa 7533, which is paired with NWA 7034 (Humayun et al., 2013). The Pb isotopic measurements they report for feldspars in Clasts 1, 4, 5, and 6 overlap with our data. Arguing from a combined set of U and Pb measurements on these clasts and others, they proposed that their Pb data represents mixing between Pb carried by feldspars, which must have been nearly free of U since 4,428 Ma, and phosphates that formed from a similar parent reservoir 1,350 Ma ago.

Taken together, Zagami and NWA 7034 highlight the spatial limitations of our experiment. In the case of Zagami, we noted that our analytical spot size was too large to observe the ~20 μm baddeleyite crystals of Zhou et al. (2013) without mixing in their neighbors. Likewise, in the case of NWA 7034, Bellucci et al. (2015) observed their pattern of Pb isotopic variations on the scale of single crystals taken from specific millimeter‐ and submillimeter‐sized clasts. Alternatively, the ensemble of our measurements may have covered a too‐small portion of the meteorite to observe significant isotopic variation, since all 200 of our measurements were within a few mm of one another. Solving these two problems simultaneously would require a much greater number of measurements of much smaller spots, and therefore much more analytical time. In this context, it bears mention that our spaceflight lasers presently under development have a pulse repetition rate of 10 kHz rather than the 20 Hz rate at which the data in this paper were acquired. Therefore, once these lasers are integrated into the instrument, we expect to be able to analyze spots nearly 500 times faster.

### 7.4 Analyses of Lunar Meteorites

#### 7.4.1 MIL 05035

**Figure 5:**
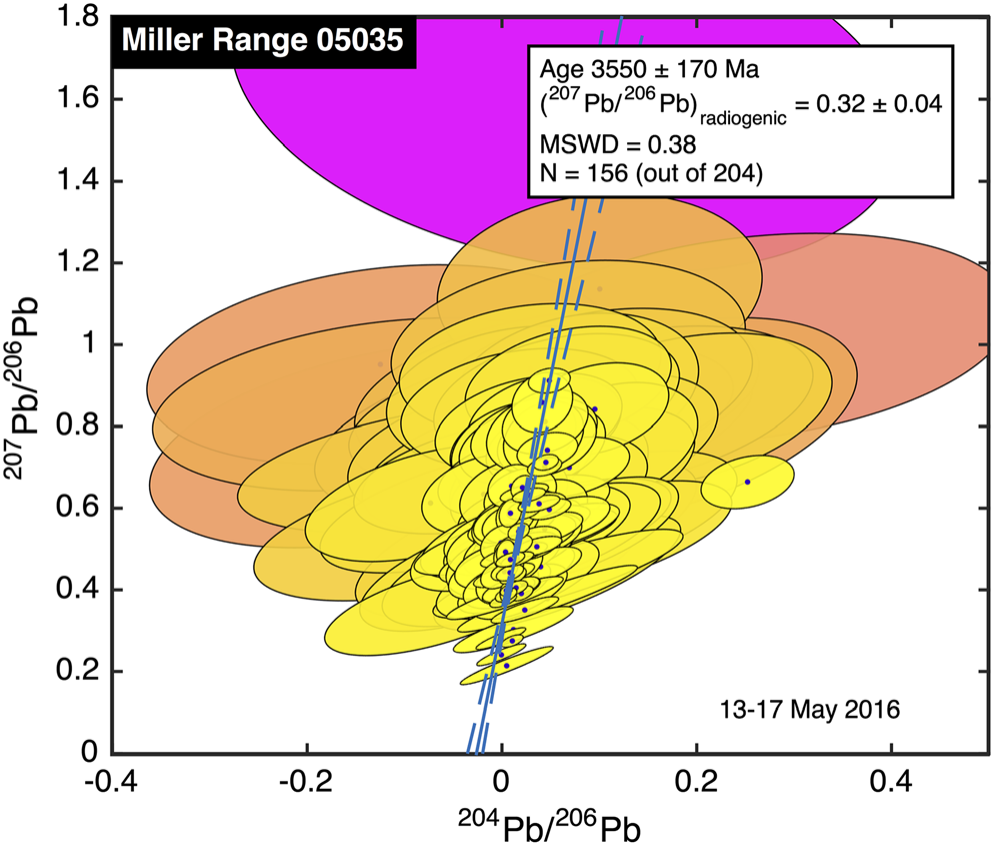
Pb‐Pb isochron diagram for MIL 05035. Though most of the 156 analyses have large uncertainties, especially in ^204^Pb/^206^Pb, because of the very low Pb concentration in this meteorite, the entire ensemble of data suffices to determine an age of 3,550 ± 170 Ma, within 2σ of the age reported by Zhang et al. (2013). Color code is described in Figure 2.

We analyzed 204 spots on MIL 05035 in a total of 14 hr over 2 days. The low Pb abundance of 0.4 ppm noted by Liu et al. (2009) made it so that even the relatively abundant Pb isotopes were below our detection limit in approximately 40% of our spots. All of the remaining 156 are illustrated in Figure 5. Only in six spots was the ^204^Pb detected with 95% confidence. This is represented graphically in Figure 5, where the error ellipses for most measurements are large, especially in the horizontal direction.

Notwithstanding the large uncertainties on individual measurements, in the ensemble of the 156 analyses with >2σ detections of ^206^Pb, ^207^Pb, and ^208^Pb, we see a definite linear trend, especially in the most precise measurements. The best fit line implies an isochron age of 3,550 ± 170 Ma, which is within 2σ of the zirconolite age measured by Zhang et al. (2010). Here we note that Zhang et al. (2010) carefully selected the single most ideal crystal for Pb‐Pb dating, whereas our approach is to eschew complex sample preparation while analyzing as much of the specimen as possible. Despite the differences in sample treatment, the ages obtained by both methods are close to one another, albeit with ours having much lower precision.

A comment is in order about the four spot analyses with the lowest ^207^Pb/^206^Pb ratios. Though all of these points are within ~1σ of the line that best fits the entire ensemble of data, the line comes closest to these four measurements at unphysical negative values for the ^204^Pb/^206^Pb ratio. Indeed, these four measurements have lower ^207^Pb/^206^Pb ratios than the pure radiogenic endmember deduced from the linear fit. It is clearly not possible for any Pb to be more‐than‐purely radiogenic; as it happens, these four spots were analyzed close to one another in time, and we suspect that a poor measurement of the standard near that time affected our calibration. We include those measurements nonetheless to illustrate that the entire ensemble of data is robust to occasional flaws of this sort.

#### 7.4.2 LAP 02205

We analyzed LAP 02205 in two separate runs: 200 spots over 44 hr in March 2016, and 128 additional spots over 20 hr in May 2016. The more abundant Pb isotopes were detected in all but one of these spots, and even ^204^Pb was detected with >2σ confidence in 189 of them.

**Figure 6:**
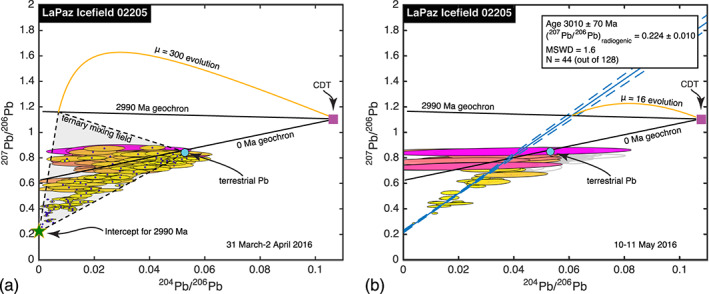
a. Pb‐Pb isochron diagram for LAP 02205, in our March 2016 run. Our data occupy a ternary mixing field defined by a radiogenic component whose age is ~2,990 Ma, terrestrial Pb contamination, and an inherited component whose composition is near the intersection of the 2,990 Ma geochron with the evolution curve for a reservoir of μ = ^238^U/^204^Pb = 300. Color code is described in Figure 2, b. Pb‐Pb isochron for LAP 02205, in our May 2016 run. Eighty‐three spot analyses had ^204^Pb/^206^Pb ratios greater than 0.04, and because they were dominated by terrestrial Pb, we excluded them from the best fit line (gray ellipses). The remaining 44 points define a linear array, and the intercept of the best fitting line implies an age of 3,010 ± 70 Ma, consistent with published ages measured with Rb‐Sr and Sm‐Nd. Color code is described in Figure 2.

In the earlier run, the Pb we detected was clearly a ternary mixture of radiogenic Pb, which we assume was inherited from the rock's crystallization on the Moon, and Pb of terrestrial isotopic composition (Figure 6a). The radiogenic endmember is consistent with an ~2,990 Ma age inferred for this meteorite using Rb‐Sr, Sm‐Nd, and Ar‐Ar (Fernandes et al., 2009; Nyquist et al., 2005; Rankenburg et al., 2007), and with the U‐Pb age of phosphates (Anand et al., 2006). The inherited component is consistent with the meteorite's crystallization at 2,990 Ma from a reservoir with a modern‐day ^238^U/^204^Pb ratio of a few hundred or more. Such high ^238^U/^204^Pb ratios are common among lunar basalts (e.g., Snape et al., 2016), in accordance with the general depletion of the Moon in volatile elements including Pb.

In the later run, there is also terrestrial contamination, but there is less evidence of the inherited Pb component (Figure 6b). Following Snape et al. (2016), we focused attention on analyses with the lowest ^204^Pb/^206^Pb ratios, which presumably are least affected by terrestrial contamination. Whereas Snape et al. (2016) identified radiogenic and inherited Pb components using the steepest left‐side boundary of the ternary data field, we simply filtered out spot analyses with ^204^Pb/^206^Pb > 0.04 (83 gray ellipses shown in Figure 6b). We find that the remaining 44 measurements lie on a linear array. The simplest interpretation of the best fit line is as a binary mixture of radiogenic Pb and a single nonradiogenic component; under this assumption, the age implied by the radiogenic Pb is 3,010 ± 70 Ma, once more consistent with the age determinations of Nyquist et al. (2005), Anand et al. (2006), Rankenburg et al. (2007), and Fernandes et al. (2009). The best fit line intersects the 2,990 Ma geochron at the evolution curve for a modern‐day ^238^U/^204^Pb ratio of 16, an uncommonly low value for lunar material. This implies that the nonradiogenic component helping to define the linear array is dominated by terrestrial Pb, in spite of our having filtered out the analyses with the most terrestrial isotopic compositions. Alternative explanations of these data are possible, since none of our analyses yielded pure radiogenic Pb, but the agreement of our inferred age with those of Nyquist et al. (2005), Anand et al. (2006), Rankenburg et al. (2007), and Fernandes et al. (2009) gives us confidence in our interpretation.

It bears mentioning that, whereas we observe significant amounts of terrestrial Pb in this meteorite, such contamination will not plague in situ analyses of unbrecciated samples on the Moon, making the interpretation of isochron ages more straightforward. Investigation of clean Apollo samples might be able to demonstrate this, but many of the Apollo samples were also contaminated with terrestrial Pb, possibly during cutting (Nunes et al., 1977).

#### 7.4.3 NWA 032

**Figure 7:**
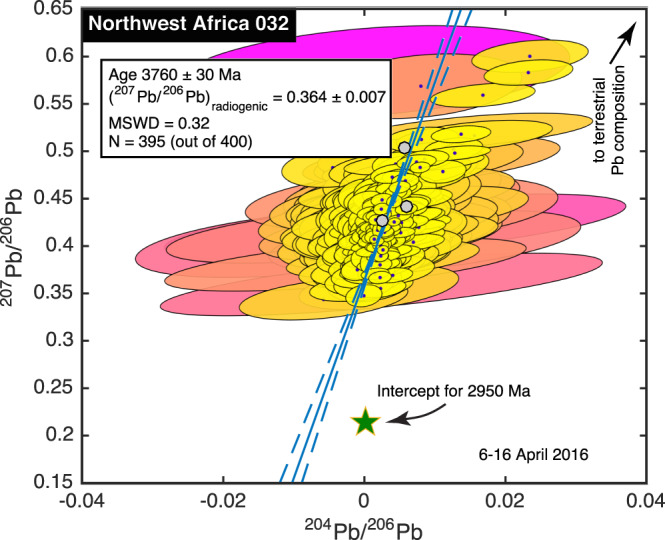
Pb‐Pb isochron for NWA 032. All of our measurements, illustrated by their 1σ uncertainty ellipses, have very low ^204^Pb/^206^Pb ratios, so the Pb we observe is nearly all radiogenic. The age determined from the best fit line is 3760 ± 30 Ma. By contrast, the Rb‐Sr and Sm‐Nd ages are about 2,950 Ma (Borg et al., 2009), and a radiogenic Pb component consistent with a 2,950 Ma age (green star) is very far from our best fit line. Color code is described in Figure 2.

We performed two runs on NWA 032 a week apart, each comprising 200 spots, and each taking about 20 hr. Data from the 395 spots in which ^206^Pb, ^207^Pb, and ^208^Pb were detected with 95% confidence are illustrated in Figure 7.

The ^204^Pb/^206^Pb ratios we measured on all spots are very low, with all values below 0.025, and 97% of them below 0.01. This suggests we are observing very radiogenic Pb. The best fit line through all the data does not pass near the terrestrial Pb composition, so there is no evidence of contamination with anthropogenic Pb. Instead, the best line appears to represent a mixture between radiogenic Pb and Pb inherited from a high U/Pb source. The ^207^Pb/^206^Pb ratio of the radiogenic endmember is 0.364 ± 0.007, implying an age of 3,760 ± 30 Ma. The isochron line intersects the 3,760 Ma geochron at the evolution curve characteristic of a reservoir with ^238^U/^204^Pb of about 50 having evolved from the initial solar system Pb composition (Tatsumoto et al., 1973). Most lunar samples appear to be derived from reservoirs with higher U/Pb ratios (Snape et al., 2016), but there is evidence for a few lunar glasses and meteorites being derived from reservoirs with ^238^U/^204^Pb ratios equal to, or even less than, the value of 50 that we deduce here (e.g., Misawa et al., 1993).

The 3,760 ± 30 Ma age that we determine for this meteorite is 800 Ma older than the Rb‐Sr and Sm‐Nd ages measured by Borg et al. (2009), and 1,000 Ma older than the Ar‐Ar age measured by Fernandes et al. (2003). To ensure that we were analyzing the correct meteorite, we recontacted the vendor, who assured us that our sample was taken from the main mass of NWA 032 (Allan Lang, personal communication, 2017). To test whether we had made an unrecognized error in our experiment, we gave part of our sample to L. Farmer at the University of Colorado, who performed three Pb analyses on it by microdrill TIMS. One measurement was without leaching, to best compare with our procedure, and after drilling, two were treated with HCL (1.5 and 4 N solutions) in case there had been any terrestrial Pb adsorbed to the sample. This acid treatment is a common technique for preparing samples for Pb isotopic analysis (e.g., Borg et al., 2005), though in some cases, terrestrial contamination persists in samples that have undergone acid leaching (e.g., Snape et al., 2019). All three measurements, shown as gray hexagons in Figure 7, overlap with our own data array (L. Farmer and E. Verplanck, personal communication, 2016, 2017). Though the three thermal ionization measurements are not sufficient on their own to constrain the age of the sample, they confirm the accuracy of the ensemble of data that we obtained. Finally, to test whether our age determination was skewed by an unrecognized, small admixture of terrestrial Pb, we filtered our data (Snape et al., 2016) to remove spots with the highest ^204^Pb/^206^Pb ratios. As we did so, the apparent age became younger (in other words, the array of our measurements is slightly concave downward). However, such tuning can, in this case, produce nearly any age: with the maximum ^204^Pb/^206^Pb ratio set to 0.0045, the apparent age was still 3,500 ± 200 Ma (with 322 points in the isochron), but with the maximum ^204^Pb/^206^Pb ratio adjusted to 0.0035, the 273 points in the isochron fit a line so nearly vertical that the age was consistent with any time in the last 3,900 Ma. We could identify no tuning parameter under which our data would require a 2,900 Ma age. These considerations give us confidence that we have accurately measured the Pb isotopic compositions of spots in NWA 032.

It might be possible to explain our Pb observations not as an isochron, but rather as a contaminant of very radiogenic lunar Pb, or even as a ternary mixture of Pb from a high‐μ lunar reservoir, the terrestrial environment, and a 2,950 Ma radiogenic component. Neither of these hypotheses are the simplest explanation of the data, but they could be tested by measuring U along with Pb, potentially in targeted phases such as zircons or phosphates, to see whether the ^204^Pb/^206^Pb decreases in inverse proportion to the ^238^U/^204^Pb ratio. Finding such a relationship would strengthen the identification of a new, much older age for NWA 032. Alternatively, if ^238^U and ^206^Pb, or ^235^U and ^207^Pb, are poorly correlated with one another, then the age that we have deduced cannot be justified. Pb‐Pb SIMS measurements might also provide insight into resolving this discrepancy.

Pending the outcome of U‐Pb or SIMS Pb‐Pb analyses, the simplest explanation of our Pb‐Pb data is that the age of NWA 032 is 3,760 ± 30 Ma and that the much younger Rb‐Sr, Sm‐Nd, and Ar‐Ar ages much represent resetting of those isotopic systems at later times. Our data do not offer insight into the kind of event that could have reset the Rb‐Sr, Nd‐Sm, and Ar‐Ar systems at 2,900 or 2,700 Ma, while leaving the Pb‐Pb system undisturbed, though plausible explanations have been offered before for similar discrepancies in other meteorites (e.g., Bouvier et al., 2009).

## 8 Conclusion

We have made the first Pb‐Pb geochronology measurements by laser‐ablation resonance ionization mass spectrometry. Compared with data acquired by other techniques, our data were obtained rapidly: From a hand‐polished, epoxy‐mounted chip of Keuhl Lake Zircon 91500, we obtained an age of 1,090 ± 40 Ma in 2 hr. Most of our analytical runs on meteorites took 20–24 hr, which is still much faster than a technique that requires chemical isolation of Pb, such as ICP‐MS or TIMS.

Like our analysis of Zircon 91500, all our tests on lunar meteorites yielded statistically meaningful ages. MIL 05035, with its bulk Pb concentration of only 0.4 ppm, nevertheless gave an isochron age of 3,550 ± 170 Ma, which is consistent at the <2σ level with a zirconolite age from Zhang et al. (2010), the only other Pb study of this meteorite. NWA 032 yielded a very precise isochron age of 3,760 ± 30 Ma, which is dramatically different from the ages determined with other isotopic systems (Borg et al., 2009; Fernandes et al., 2003). Identifying the source of this discrepancy will be important for understanding the geologic history of the Moon and the fidelity of the Pb‐Pb dating system for lunar samples. The Pb in LAP 02205 was dominated by terrestrial contamination, but filtering out the most contaminated spots from one of our runs yielded an isochron line corresponding to an age of 3,010 ± 70 Ma, indistinguishable with Rb‐Sr and Sm‐Nd ages (Nyquist et al., 2005; Rankenburg et al., 2007). The data from our other run were consistent with this age as well, but in the presence of ternary mixing of radiogenic, inherited, and terrestrial Pb, we were unable to make more than a consistency check. However, it bears mention that terrestrial contamination will not pose the same challenge for in situ analyses on the Moon.

Two challenges made it more difficult for us to extract useful geochronological information from the Martian meteorites we studied. First, Martian meteorites generally have low U/Pb ratios, and therefore, most of the Pb in the minerals they contain is nonradiogenic. This means that an isochron line would need to be extrapolated to a quite distant radiogenic endmember composition in order to determine an age, and it also implies that even modest amounts of contaminant Pb can overwhelm the radiogenic Pb and give rise to spurious age interpretations. Second, Mars appears to have a reservoir of such a contaminant on its surface (Bellucci et al., 2016), so even in situ experiments might need to interpret Pb‐Pb ages in the presence of ternary mixing.

A common approach when modeling ternary mixtures of Pb is to adopt the age of the sample as determined by Rb‐Sr or another radioisotopic system (Bellucci et al., 2016; Borg et al., 2005). This is one reason why the ability to determine ages with multiple radioisotopic systems would be an important asset for an in situ dating experiment. Our resonance ionization mass spectrometer can easily accommodate multiple decay systems by incorporating lasers tuned to resonances in Rb and Sr as well as Pb. For the wavelengths required for Pb, custom fibers may be possible to develop; however, better alternatives may exist either by nonlinear mixing of upconverted light from Er or Tm and Yb gain media, or by developing a smaller and more efficient diamond raman laser (e.g., Chrysalidis et al., 2019). The high repetition rate of the lasers we are developing for spaceflight means that we will be able to acquire data 500 times faster than we do today. This factor of 500 will make it feasible to examine many more points on a lunar or Martian specimen, as will be necessary to sample the accessory minerals that evidently carry the clearest signatures of radiogenic Pb (Zhou et al., 2013). Moreover, integrating optics to more tightly focus the ablation laser will allow us to analyze spots small enough to probe finer‐grained minerals, such as the baddeleyite carriers of radiogenic Pb in Zagami, without diluting them with Pb from neighboring grains.

Among the improvements we have made to CODEX in the time since we made the Pb measurements reported here, changing the geometry of the source region of the mass spectrometer proved especially important for reducing baseline noise (Anderson et al., 2020). By more effectively excluding ablated plasma from the mass spectrometer, we have lowered the baseline noise by a factor of ~3, making it easier to detect small signals, like those we observed in the present work from ^204^Pb. It will be important to retain this new geometry in future CODEX tests of Pb‐Pb dating. In addition, future CODEX tests will couple resonance ionization mass spectrometry measurements of isotopic abundances of specific elements with laser ablation mass spectrometry measurements of generic elemental abundances, in order to complement our isotopic dates with information about petrologic context (Anderson et al., 2014; Foster et al., 2016).

The Pb‐Pb dating results we have presented, with 70 Ma uncertainty on LAP 02205 and 30 Ma uncertainty on NWA 032, are sufficiently precise to address both the billion‐year uncertainty in crater‐count chronologies of the inner solar system and the absolute ages of lunar basis like Nectaris. Results from such measurements, with their implications for dynamics of asteroid‐belt populations, ages of terranes on the Moon and Mars, duration of the Martian habitability era, and the epoch of the first life on Earth, will inform the next half century of solar system science. This work demonstrates that in situ isochron dating using multiple decay systems is within reach for a future lander or rover missions to another planetary body.

## Data Availability

The data described in this paper is archived and available for free download using a standard web browser (at https://commons.colgate.edu/physics_facpub/1/).
